# An overview of the quality assurance programme for HIV rapid testing in South Africa: Outcome of a 2-year phased implementation of quality assurance program

**DOI:** 10.1371/journal.pone.0221906

**Published:** 2019-09-26

**Authors:** Selamawit Alemu Woldesenbet, Mireille Kalou, Dumisani Mhlongo, Tendesayi Kufa, Makhosazana Makhanya, Adeboye Adelekan, Karidia Diallo, Mahlatse Maleka, Beverley Singh, Bharat Parekh, Amanda Mohlala, Peter T. Manyike, Tim J. Tucker, Adrian J. Puren

**Affiliations:** 1 Center for HIV and STI, National Institute for Communicable Diseases, Johannesburg, South Africa; 2 School of Public Health, University of the Witwatersrand, Johannesburg, South Africa; 3 International Laboratory Branch, Division of Global HIV and Tuberculosis, Center for Global Health, Centers for Disease Control and Prevention, Atlanta, Georgia, United States of America; 4 National Clinic Laboratory Interface programme, National Health Laboratory Service, Johannesburg, South Africa; 5 Laboratory Branch, Centers for Disease Control and Prevention South Africa, Pretoria, South Africa; 6 Academic Affairs, Research and Quality Assurance National Health Laboratory Service, Johannesburg, South Africa; 7 Strategic Evaluation, Advisory and Development (SEAD) Consulting, Cape Town, South Africa; 8 School of Public Health and Family Medicine, University of Cape Town, Cape Town, South Africa; 9 Virology Department, University of the Witwatersrand, Johannesburg, South Africa; University of Ghana College of Health Sciences, GHANA

## Abstract

**Objective:**

This is the first large-scale assessment of the implementation of HIV Rapid Test Quality Improvement Initiative in South Africa.

**Methods:**

We used a quasi-experimental one group post-test only design. The intervention implemented starting April 2014 comprised health-care worker training on quality assurance (QA) of HIV rapid testing and enrolment of the facilities in proficiency testing (PT), targeting 2,077 healthcare facilities in 32 high HIV burden districts. Following the intervention, two consecutive rounds of site assessments were undertaken. The first, conducted after a median of 7.5 months following the training, included 1,915 facilities that participated in the QA training, while the second, conducted after a median of one-year following the first-round assessment included 517 (27.0%) of the 1,915 facilities. In both assessments, the Stepwise-Process-for-Improving-the-quality-of-HIV-Rapid-Testing (SPI-RT) checklist was used to score facilities’ performance in 7 domains: training, physical facility, safety, pre-testing, testing, post-testing and external quality assessment. Facilities’ level of readiness for national certification was assessed.

**Result:**

Between 2016 and 2017, there were four PT cycles. PT participation increased from 32.4% (620/1,915) in 2016 to 91.5% (1,753/1,915) in 2017. In each PT cycle, PT results were returned by 76%–87% of facilities and a satisfactory result (>80%) was achieved by ≥95% of facilities. In the SPI-RT assessment, in round-one, 22.3% of facilities were close to or eligible for national certification—this significantly increased to 38.8% in round-two (P-value<0.001). The median SPI-RT score for the domains HIV pre-testing (83.3%) and post-testing (72.2%) remained the same between the two rounds. The median score for the testing domain increased by 5.6% (to 77.8%).

**Conclusion:**

Facilities performance on the domains that are critical for accuracy of diagnosis (i.e. pre-testing, testing and post-testing) remained largely unchanged. This study provided several recommendations to improve QA implementation in South Africa, including the need to improve routine use of internal quality control for corrective actions.

## Introduction

HIV is a major public health problem in sub-Saharan African countries, which are home to more than half of all people living with HIV (PLHIV)[1]. South Africa had 270,000 new HIV infections and 7.2 million PLHIV in 2017 [1]. According to the Joint United Nations Programme on HIV/AIDS (UNAIDS) 2017 report, an estimated 10% of PLHIV in South Africa do not know their HIV status, and many (40%) who know their HIV status either do not initiate treatment immediately, or default after initiating treatment [1].

HIV testing services (HTS) play a critical role in identifying and linking HIV infected individuals to treatment services. In an effort to increase the number of PLHIV initiated on antiretroviral treatment (ART), on average about 10 million HIV tests are performed annually in South Africa using rapid diagnostic tests (RDTs) [2]. Given the large scale of tests performed annually, ensuring accuracy of test results is a priority. Reports from various studies in the country indicate false positive and false negative rates may be as high as 2.4% and 8.9%, respectively [3, 4]. These error rates could result in thousands of misdiagnoses on a yearly basis, with devastating impact on both programmes and individuals [2, 5, 6]. HIV testing using World Health Organization (WHO) qualified RDTs is highly accurate when standard quality assurance (QA) processes are followed [7]. Often the low performance of RDTs is due to inadequate implementation of QA processes, use of poorly performing test kits or testing algorithm, acute infections, and cross-reactivity [3, 5, 8–15].

International development partners including the United States (U.S.) Centers for Disease Control and Prevention (CDC) and WHO recognised the need to support countries in the implementation of QA programmes [16]. Assessments were conducted to gather lessons learnt from the country programmes which led to further guidance [16]. Subsequently, the need for QA activities was re-emphasised in for example, President’s Emergency Plan for AIDS Relief (PEPFAR) country operational plans (COP) and in the recent WHO HTS guidelines [17, 18].

In 2016, South Africa with support of PEPFAR, introduced HIV Rapid Test Continuous Quality Improvement Initiative (RTCQI), which aimed to improve the quality management system (QMS) for HIV rapid testing at HIV testing site level. The RTCQI programme built on existing national programmes consisted of the following components: (1) policy engagement to enable a framework for policy development; (2) strengthening local capacity to increase uptake of QMS at site level and cadres of qualified testers through training toward national certification; (3) proficiency testing (PT) programs (4) increasing the uptake of standardized register and data collection tools; and (5) post market surveillance of RDTs.

This paper outlines QA processes implemented and describes the outcomes of the two-year implementation of the QA intervention in 2,077 testing facilities across 32 high HIV burden districts in South Africa.

## Methods

### Study design

The study used a quasi-experimental one group post-test only design. The intervention consisted of four QA interventions implemented starting April 2014: (i) QA training, (ii) internal quality control (IQC), (iii) PT and (iv) strengthening post-market surveillance targeting 2,077 public facilities, selected from 32 high HIV burden districts.

QA training started in April 2014. Following the roll out of the QA training, two consecutive site assessments were conducted using the Stepwise-Process-for-Improving-the-quality-of-HIV-Rapid-Testing (SPI-RT) checklist (S1 and S2 Files). The first round assessments were conducted between the 18^th^ of April 2015 and 13^th^ of December 2017; after a median of 7.5 months (inter-quartile range, IQR: 4.0–10.0 months) following the QA training (S1 Fig). After a median of one-year (IQR: 11 months–15 months) following the first-round assessment, second round assessments were conducted. Both assessments targeted all 2,077 facilities that participated in the QA training and aimed to monitor progress and sustained implementation of RTCQI activities at those facilities.

### Facility selection

Twenty-seven (84.4%) of the 32 districts targeted for RTCQI implementation were PEPFAR-supported districts, and 5 additional high-burden districts that were partially supported by PEPFAR were included. In the 27 PEPFAR-supported districts, facilities were selected if they had at least 500 patients already initiated on ART or were ranked at the top for contributing to 90% of the ART, prevention of mother-to-child transmission (PMTCT) or HTS coverage nationally. In the five districts, facilities were selected if they ranked at the top for contributing to 80% of the national ART coverage or if they were within the top facilities that contributed to the first 25,000 of the ART coverage within their respective districts [19].

### Description of interventions

The QA programme in South Africa is led by the National Department of Health (NDoH). The HIV RTCQI plan, endorsed by the NDoH in 2014, outlined a package of QA activities identified to strengthen the priority areas that needed improvement. Details of these priority areas and the roles and responsibilities of key stakeholders for implementing these priority areas are listed under S1 Table.

Under the National Institute of Communicable Diseases (NICD)/National Health Laboratory Service (NHLS) leadership, the following four QA interventions were implemented to improve QA practices in the target facilities: (i) training of health-care workers on HIV RDTs QA, (ii) use of internal quality control (IQC), (iii) participation in PT programme, and (iv) strengthening post-market surveillance of RDTs.

### Training

QA training was rolled out in eight of the nine provinces (excluding Northern Cape) using various training approaches: (1) direct training of facility staff by NICD, (2) Regional Training of Trainers (TOT) using RTCQI training package which was subsequently rolled out to national and district level, (3) national RTCQI TOT and (4) district level RTCQI training.

#### 1. Rapid testing direct QA training of facility staff (2014 and 2015)—Prior to the RTCQI launch)

Prior to the introduction of the RTCQI programme, NICD ran a QA programme that included direct training of facility staff and test providers at the provincial, district and sub-district levels. Prior to 2016, facility staff were trained directly by NICD trainers using this approach. A two-day long training was provided at each training site targeting facility staffs and test providers. The training comprised 16 modules that were directly adapted from the WHO QMS training package [20]. Topics covered and methods used during training were similar between the direct training of facility staff and the RTCQI training described below. The outcome of the training was evaluated using pre- and post-training assessment questionnaires but pre- and post- training assessment results were not available for analysis.

#### 2. Regional RTCQI training

The RTCQI programme was officially launched on 31 August 2015 during a two-week long African regional training session in Johannesburg, South Africa. Nineteen delegates from South Africa were trained as master trainers in the regional training conducted by a team from CDC-Atlanta. Five months after the regional training (on 22 February 2016), the delegates initiated the national roll-out of the RTCQI programme in South Africa. The TOT approach was used to cascade the training down to the facility level.

#### 3. National RTCQI training of trainers (TOT) (2016 and 2017)

A week-long (22–25 February 2016) national TOT on QA targeting implementing partners, and provincial and district trainers was facilitated by NICD. Ninety-four participants from provincial departments of health, non-governmental organisations, and academic institutions attended the training. The participants had the following roles in their respective provinces or districts: 20.2% (19/94) were management staff, 44.7% (42/94) were department of health program coordinators, and 35.1% (33/94) were trainers or mentors. The aim of the training was to empower mid- to high-level programme staff, quality officers, and trainers overseeing testing facilities, and create a pool of master trainers (through TOT) who would be responsible for cascading the QA training to the district and facility levels. The comprehensive training package developed by the CDC International Laboratory Branch was revised by the NICD to adapt it to the South African context, and was used for the training. The training programme was comprised of 11 modules, which were delivered during classroom sessions using hands-on and instructor-led activities. These activities included laboratory practical sessions on testing IQC and PT samples, completing standardised HIV testing registers, data review, and analysis. The training was co-facilitated by the NICD, master trainers from implementing partners, and the NDoH. Training outcome was assessed using pre- and post-training assessment questionnaires. Of the 94 participants, 82 (87.2%, 82/94) met the required standards for the post-training evaluation (i.e., scored >80% in the post-training evaluation). On the last day of the workshop, provinces presented their draft training plans for cascading the training to the district level.

#### 4. Roll-out of district-level training

Master trainers trained in the national TOT were responsible for cascading the training down to the district, sub-district, and facility levels. The training was organised at the district and sub-district levels, targeting in particular sub-district programme coordinators and trainers, facility managers, and facility staff who provide HIV testing in the 32 high-burden districts. At least one training per district was observed by assessors from NICD. Assessors completed an observation checklist and scored the master trainers on their performance. The assessment demonstrated that, of the 96 trainers assessed, 60.4% (58/96) were ready to conduct training, and 39.6% (38/96) still needed more development as master trainers. After the observation, feedback on their performance was given to the participants. It was the responsibility of the district training team to ensure that those who did not demonstrate adequate training skills received the necessary training before continuing to train in the subsequent district-level training sessions. No further follow-up was made to document improvement in training skills among those who had demonstrated inadequate training skill in the first observation. The pre- and post-training assessment was completed by participants after each district-level training session, but results were not available for analysis. After each training session, district master trainers reported on the number of testers trained, and the duration of the training.

### Proficiency testing scheme

Proficiency testing for HIV rapid testing was rolled out in eight of the nine provinces (excluding Northern Cape) by NHLS. During RTCQI training, participants were trained on the testing procedure of the PT panels and the completion of response forms following testing of PT panels. All facilities that received RTCQI training were expected to enrol in the PT programme. Between 2016 and 2017, there were four PT cycles and each cycle consisted of facilities receiving six blinded panel members (S2 Table). A PT result form (S3 File) sent with each panel, was completed by testers and returned to the NHLS PT programme coordinator. The facilities participating in the PT programme were given five weeks to submit results (completed forms) before the closing date. Submission was done by either fax or email. Provinces assisted the facilities with limited resources to submit results on time. Each facility was assigned a unique identification code in order to maintain confidentiality.

At the time of the PT implementation, the testing algorithm in South Africa, followed the WHO serial 2-test algorithm. Prior to 2017, the nationally recommended algorithm combined Advanced Quality Rapid Anti-HIV (1&2) for first-line and ABON HIV 1/2/O Tri-Line for second-line. In 2017, the recommended algorithm included wide range of test kits listed under S3 Table.

The following outcome indicators were reported for PT:

**Enrolment in PT**: percentage of facilities’ PT panels distributed over the total number of facilities that participated in SPI-RT assessment. This was reported, disaggregated by year and cycle (2016 first cycle, 2016 second cycle, 2017 first cycle, and 2017 second cycle).

**Participation in PT**: total number of facilities returned PT results over the total number of facilities that received a PT panel in each cycle.

**Satisfactory result**: Percentage of facilities with satisfactory results (>80%) in each cycle. The NHLS PT provider defined the acceptable percentage range for successful performance as 80%. Two marks were given to each of the six panel members tested if the test result (HIV status) was correct, a zero mark was given if the test result was reported incorrectly, and one mark was given for an inconclusive/discrepant result between first and second line test. The maximum achievable score per PT round was 100% (12/12). A percentage score below 80% was regarded as being unacceptable and therefore corrective actions needed to be applied for these facilities [21–23].

### Internal quality control (IQC)

A total of 10,136 IQC panels of 10 serum samples of known HIV status were distributed between 2014 and 2017. Samples were confirmed as either HIV-negative or HIV-positive through reference laboratory testing (using enzyme immunoassays, blotting, and RDTs). All 32 districts participated at least once in the IQC scheme. The distribution of samples followed the same scheme as for the PT. The IQC results were documented in a log sheet or clinic HTS register, but no data extraction was done to analyse the outcomes of the IQC. Therefore, this study does not report on the outcome of IQC implementation.

### Strengthening the post-market surveillance

Since 2009, the NICD implemented compulsory lot verification testing as part of active post-market surveillance of HIV RDTs. New lots of HIV RDTs of either first-line or second-line test devices/kits were submitted to the NICD prior to release to testing facilities for verification. A limited number of test devices were selected and tested against different characterised panels to ensure that the specification criteria for lot verification determined by the NICD were met. On completion of the lot verification testing, a report on the performance of the lot and outcomes was submitted to the supplier for onward submission to the manufacturer per tender requirements and NDoH.

### Post-intervention assessments

Assessors from NICD (in round-one) and from Strategic Evaluation, Advisory and Development (SEAD) Consulting (in round-two) conducted the two rounds of post-intervention assessments using the SPI-RT version 2 and version 3 checklists (S1 and S2 Files). The SPI-RT version 2 checklist had eight domains listed in S4 Table and was revised in 2016, resulting in changes to the number of questions/ items by domain, as listed in S4 Table.

Version 2 of the checklist was used at 106/1,915 facilities during the round-one assessments. The remaining 1,809 facilities in round-one, and all round-two sites, were assessed using version 3 of the checklist.

#### Outcomes of the assessments

The following outcomes of the post intervention assessments were measured: i) overall scores on the SPI-RT checklist, determined as a total of the seven domain scores and expressed as raw totals and also as percentages of the highest possible scores (S4 Table); ii) domain- or area-specific scores; and iii) HIV rapid testing QA implementation levels, which represented levels of HIV rapid test QA programme implementation in readiness for national certification. To determine the QA implementation level, the overall percentage scores were categorised by province into levels: level 0 to level 4. The levels were determined from the total SPI-RT percentage scores, as follows: total percentage score ≤40% = level 0 (needs improvement in all areas and immediate remediation), 41%–59% = level 1 (needs improvement in specific areas), 60%–79% = level 2 (partially ready for national site certification), 80%–89% = level 3 (close to national site certification) and ≥ 90% = level 4 (eligible for national site certification).

### Data entry and analysis

Data collected from each facility using the SPI-RT checklist were uploaded into an Open Data Kit (ODK) software system (University of Washington, Washington DC., USA https://opendatakit.org/), and exported to Excel (Microsoft cooperation, Washington DC., USA). The PT and training data were captured directly into Excel. All three types of data (PT, training, and SPI-RT data) were subsequently exported into Stata 14 (Stata Corporation, College Station, Texas, USA) for analyses. Descriptive statistics were used to describe the distribution of facilities by province. The median and interquartile ranges (IQR) of the total scores and the percentage scores were determined for the overall data set, by SPI-RT domain, province, and site assessment round. The number and proportion of facilities at the different levels of QA implementation were determined by province and assessment round and the chi^2^ test used to test for an association between assessment round and implementation level. Ranksum and Kruskall–Wallis tests were used to identify significant differences in the distribution of scores between between round-one and two site assessment results and by province.

## Results

### Description of general characterises of the testing facilities

Between the 3rd of April 2014 and the 14^th^ of September 2017, nationally, 2,077 PEPFAR-supported facilities received training on HIV rapid testing QA processes (Table 1 and S2 Fig). One thousand nine hundred fifteen (92.2%) of the 2,077 facilities were visited for the first-round assessment. More than half (52.0%) of the first-round assessments were completed in 2015 and 2016, and covered eight of the nine provinces (excluding Northern Cape) (S2 Fig). Less than 10% of the 2,077 facilities that participated in training could not be assessed during the first assessment (n = 128) or had missing or inaccurate assessment data that could not be included in this analysis (n = 34).

**Table 1:** Distribution of HIV testing sites by province enrolled in RTCQI in South Africa, 2014–2017.

Province	Number of sites that received QA training (%)* (03 Apr 14–14 Sep17)	Number of sites assessed in Round-one assessment (%)** (18 Apr 15–13 Dec17)	Number of sites assessed in Round-two assessment (%)** (24 Nov16–29 Sep17)
**Eastern Cape**	490 (57.9)	423 (22.1)	61 (11.8)
**Free State**	102 (36.6)	94 (4.9)	24 (4.6)
**Gauteng**	302 (77.4)	297 (15.5)	90 (17.4)
**KwaZulu-Natal**	463 (58.8)	449 (23.4)	186 (36.0)
**Limpopo**	180 (31.2)	171 (8.9)	0
**Mpumalanga**	255 (73.9)	246 (12.8)	83 (16.1)
**North West**	231 (61.3)	225 (11.7)	73 (14.1)
**Northern Cape**	0	0	0
**Western Cape**	54 (14.3)	10 (0.5)	0
**Total**	**2,077 (49.5)**	**1,915 (100)**	**517 (100)**

* Denominator is total number of sites in the province, as follows: Eastern Cape = 846, Free State = 279, Gauteng = 390, KwaZulu-Natal = 787, Limpopo = 577, Mpumalanga = 345, North West = 377, Northern Cape = 0, Western Cape = 378, and Total = 3,979

**Denominator is total number of sites assessed in the given round

The round-two assessment covered 517 (27.0%; 517/1,915) facilities assessed in round-one (99.0% of the 517 facilities were assessed in 2017) (Table 1). The remaining 1,560 facilities had not yet been visited for round-two assessment at the time of this data analysis.

### Characteristics of the facilities

More than two-thirds of the facilities assessed in both rounds were primary health-care clinics, and >90% offered HTS (<10% of facilities provided PMTCT, provider initiated counselling and testing (PICT), or ART services). A median of four testing providers per facility provided HTS. The median average monthly testing volume was 189 (IQR: 44–320) and 160 (IQR: 80–300) in round-one and round-two, respectively.

### Training coverage

All selected 2,077 public facilities, representing 49.5% of public facilities nationally, received training on QA. Training date was available for 1,919 of the 2,077 facilities trained. QA training started in 2014 in two provinces (Mpumalanga and North West) with the majority (65.3%, 1,253/1,919) of training undertaken in 2016 (53.4%, 1,024/1,919) and 2017 (11.9%, 229/1,919) after the RTCQI launch (TOT approach used) (S2 Fig). Just above a third of facilities were trained in 2014 (6.0%, 116/1,919) and 2015 (28.7%, 550/1,919) (NICD directly trained testing providers in these facilities) (S2 Fig).

A total of 2,715 health workers were trained. Coverage of training varied by province. The highest training coverage was observed in Gauteng (77.4%, 302/390) and Mpumalanga (73.9%, 255/345)—health workers from >70.0% (557/735) of all public facilities in the two provinces received QA training (Table 1). Coverage of training was low in the Western Cape, as most facilities in the province are non-PEPFAR supported facilities and thus were not selected for the QA training.

### PT programme

#### PT participation rates

Overall, 95.2% (1,824/1,915) of facilities participated at least once in PT either before or after their assessment. More than a third (39.2%, 751/1,915) of the facilities assessed in round-one and 83.0% (429/517) of facilities assessed in round-two had participated in at least one PT cycle at the time of their assessment.

**Fig 1:**
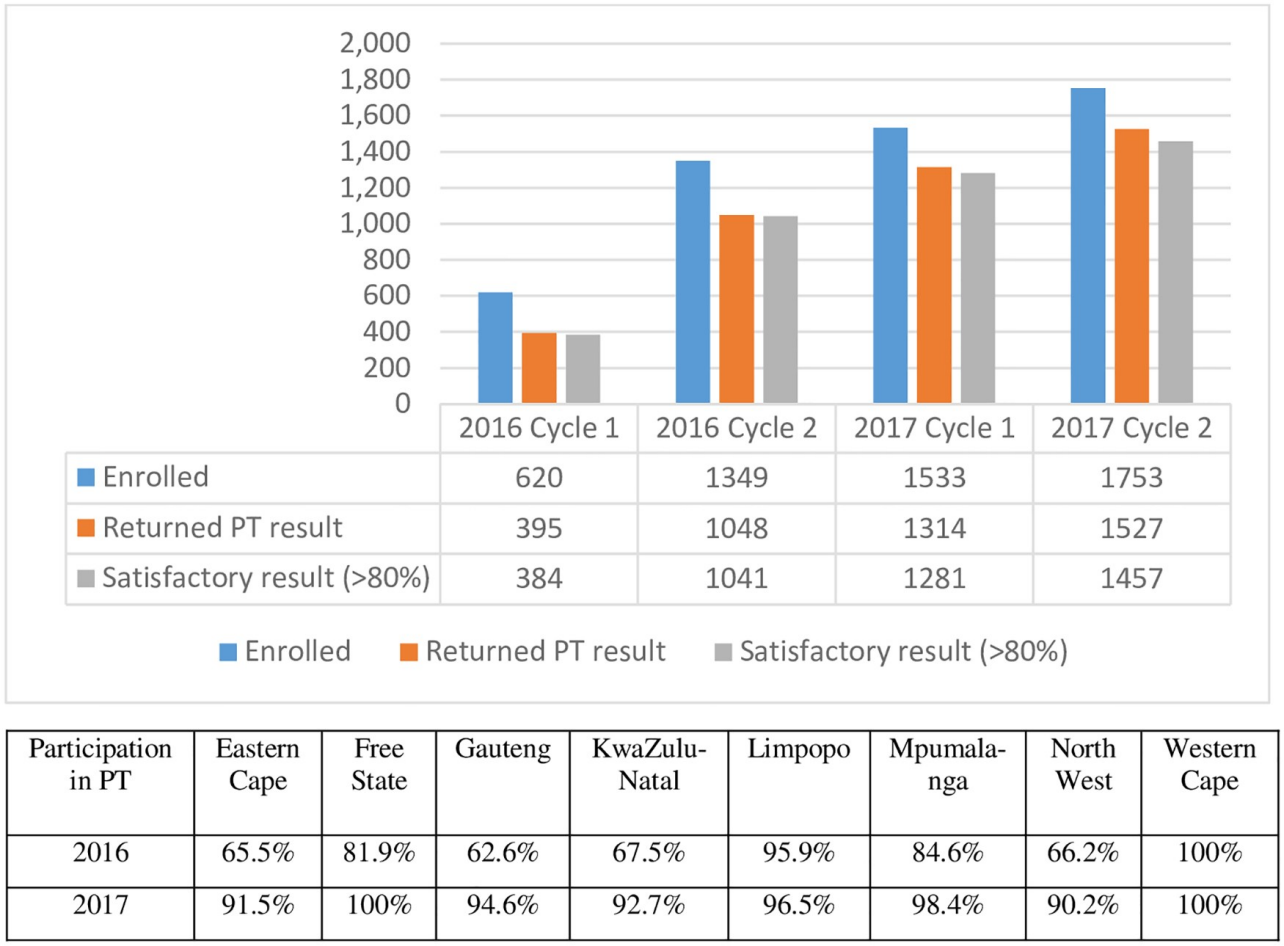
Results of proficiency testing (PT) scheme in 2016 and 2017, South Africa.

During 2016, Gauteng had the lowest PT participation rate (62.6%, 186/297), followed by Eastern Cape (65.5%, 277/423) and North West (66.2%, 149/225). In 2017, the lowest PT participation rate was observed in North West, at 90.2% (203/225) (Fig 1).

In 2016, 620 (32.4%, 620/1,915) facilities from eight provinces participated in PT cycle 1 and 1,349 (70.4%, 1,349/1,915) facilities participated in PT cycle 2 (Fig 1). In 2017, there was a significant increase in the participation rate to 80.1% (1,533 facilities/1,915) in the first cycle and 91.5% (1,753/1915) in the second PT cycle across eight provinces (excluding Northern Cape).

#### PT result return rate and performance

**Fig 2:**
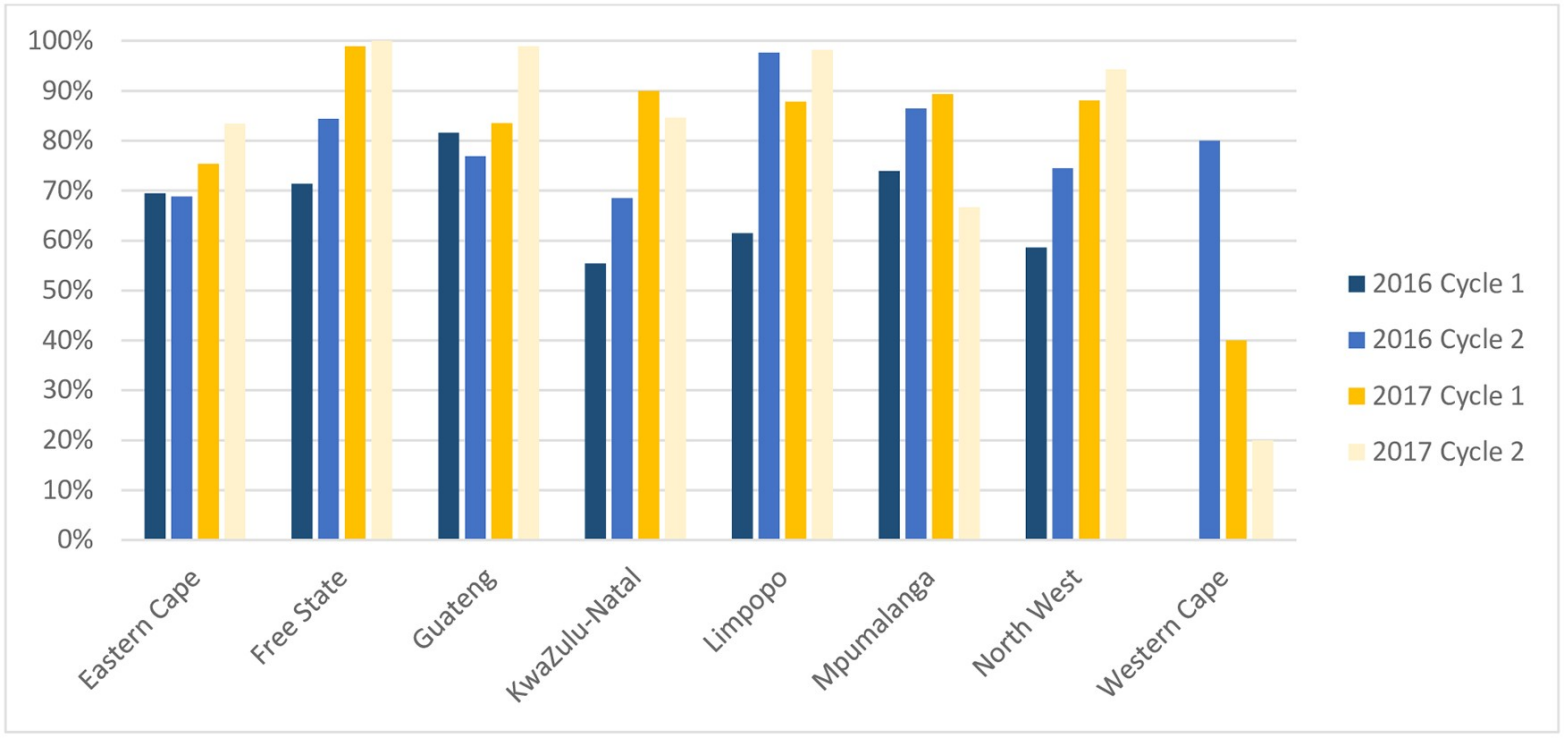
Proficiency testing (PT) programme result return rate by province, 2016–17, South Africa.

Result return rate varied by cycle (2016 cycle 1: 63.7%, 395/620; 2016 cycle 2: 77.7%, 1,048/1,349; 2017 cycle 1: 85.7%, 1,314/1,533; 2017 cycle 2: 87.1%,1,527/1,753). The result return rate improved from 2016 to 2017 in five of the eight provinces (except Mpumalanga, Western Cape, and KwaZulu-Natal) (Fig 2). Satisfactory results (> 80.0% score) were achieved by ≥95.0% of facilities in each round (Fig 1).

#### Test kits used for PT scheme

In the first three cycles (2016 cycle 1 and 2, and 2017 cycle 1), the national testing algorithm included Advanced Quality Rapid Anti-HIV (1 and 2) for first-line and ABON HIV 1/2/O Tri-Line for second-line (confirmatory) testing. In the three cycles, 83.5%–90.4% (330/395 in round 1 to 947/1,048 in round 3) of facilities used the recommended test for both first-line and second-line (S5 Table). A few facilities (≤0.3% ~1–3 facilities) deviated from the national algorithm (S5 Table).

In 2017, in PT cycle 2, although a new testing algorithm was recommended (S5 Table), most provinces still used the outdated testing algorithm (Advanced Quality [78.3%, 1,195/1,527] and ABON [76.2%, 1,163/1,527]). In most provinces, as a result of overstock of test kits purchased in a previous tender, facilities were required to use the test kits purchased for the previous testing algorithm before transitioning to the new testing algorithm.

### Site assessments

#### First-round site assessments

The median percentage score for the facilities assessed in the first-round was 70.3% (IQR61.3%– 78.1%) (Table 2). Facilities attained the lowest scores in the domains for training and certification (median percentage score: 40%) and external quality assessment (EQA) (median percentage score: 37.5%). Facilities achieved the highest score in the domains safety (median percentage score: 90.9%) and Physical facility (median percentage score: 90.0%).

**Table 2:** Distribution of median scores and percentage scores by domain among sites assessed in round one (N = 1,915), between 18^th^ of April 2015 to 13^th^ of December 2017, SPI-RT assessment, South Africa.

Domain	Median score (IQR)	Median score (%) (IQR)
**Training and certification**	4.0 (2.5–5.5)	40.0 (25.0–55.0)
**Physical facility**	4.5 (4.0–5.0)	90.0 (80.0–100.0)
**Safety**	10.0 (8.5–11.0)	90.9 (77.3–100.0)
**Pre-testing phase**	10.0 (9.0–11.5)	83.3 (75.0–95.8)
**Testing phase**	6.5 (5.0–8.0)	72.2 (55.6–88.9)
**Post-testing phase**	7.5 (6.0–8.0)	83.3 (66.7–88.9)
**EQA**	3 (1.5–6.0)	37.5 (18.8–87.5)
**Overall score**	**45.0 (39.0–50.0)**	**70.3 (61.3–78.1)**

Note: the highest possible score for each domain is as follows: personnel training and certification = 10; physical facility = 5; safety = 11; pre-testing phase = 12; testing phase = 9; post-testing phase = 9; and EQA = 8.

IQR: inter-quartile range

**Fig 3:**
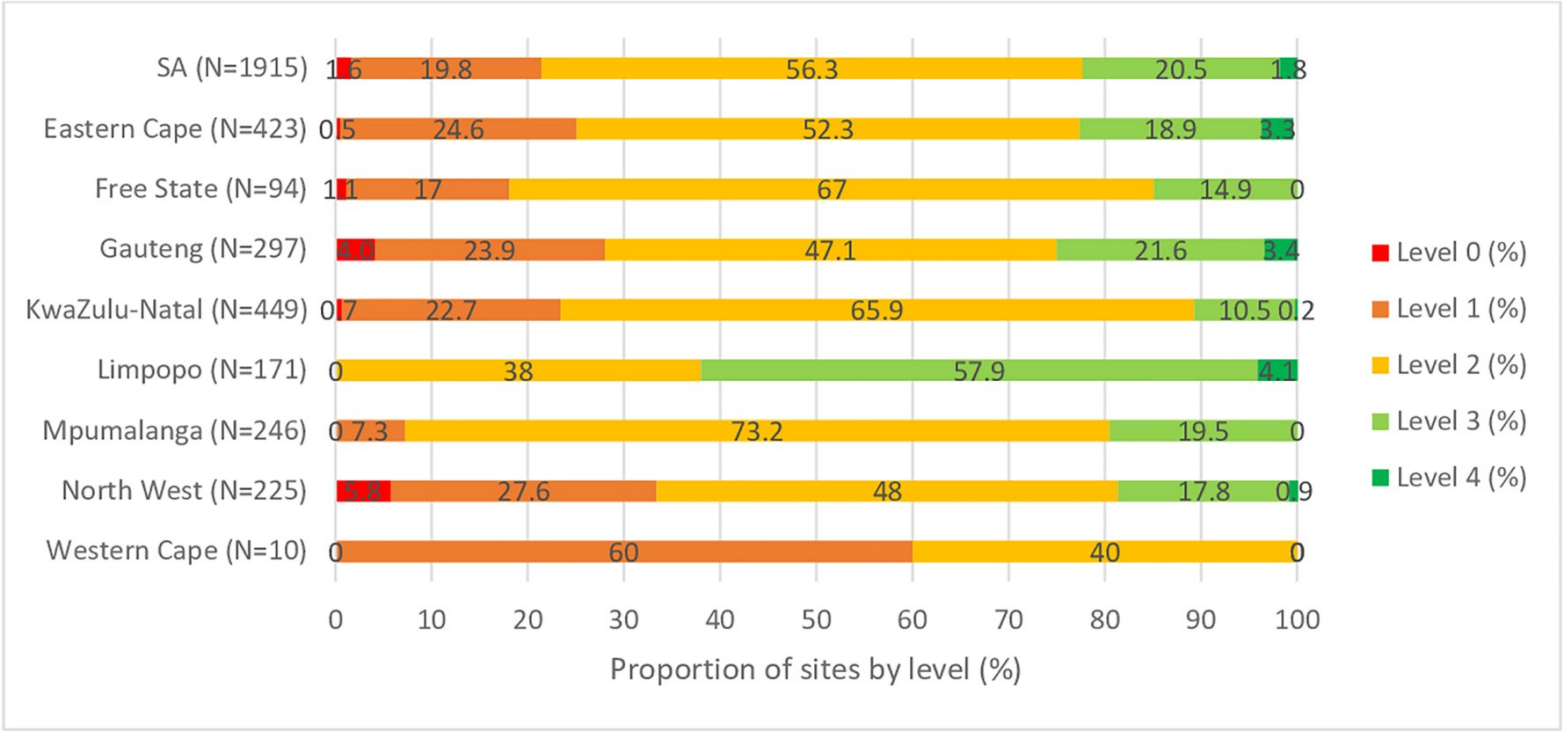
Distribution of HIV testing facilities by performance levels across 32 priority districts of South Africa in assessment round-one (N = 1,915) conducted between 18th of April 2015 to 13th of December 2017, SPI-RT assessment, South Africa.

Fig 3 shows the number of facilities classified by implementation level. More than 75% (1,488/1,915) of facilities assessed were at level 2 or below (with 21.4% of facilities requiring improvement either in all areas [1.6%, 31/1,915] or in specific areas [19.8%, 379/1,915]). A fifth of facilities (20.5%, 392/1,915) were close to national certification (level 3) and only 1.8% (34/1,915) were eligible for national facilities certification (level 4).

Facilities in Limpopo performed relatively well compared with other provinces; 62.0% (106/171) of facilities in Limpopo were at levels 3 and 4, and 38.0% (65/171) were at level 2. The lowest-performing provinces were North West [33.3% (75/225) of facilities were at level 1 or below], Gauteng [27.9%, (83/297) of facilities were at level 1 or below], and Eastern Cape [25.1%, (106/423) of facilities at level 1 or below]. Western Cape also had 60.0% (6/10) of facilities at level 1; however, the number of facilities assessed in the province were small: 10 in total.

#### Second-round site assessments

Among 517 facilities that participated in both the first- and second-round assessments, the overall median percentage score increased significantly (p value from Ranksum test = 0.001) from 64.1% (IQR: 56.3–70.3%) in the first-round assessment to 76.6% (IQR: 68.8–82.8%) in the second-round assessment (Table 3). The improvement in round-two was mainly a result of the substantial improvement observed in both the EQA, as well as training and certification domains.

**Table 3:** Distribution of median scores by assessment round and domains assessed, SPI-RT assessment, 2015–2017, South Africa (N = 517).

Domain	Round-one		Round-two	
	Median score (IQR)	Median score (%) (IQR)	Median score (IQR)	Median score (%) (IQR)
Training and certification*	3.0 (2.0–4.0)	30.0 (20.0–40.0)	5.0 (4.0–6.0)	50.0 (40.0–60.0)
**Physical facility**	4.5 (4.0–5.0)	90.0 (80.0–100.0)	5.0 (4.0–5.0)	100.0 (80.0–100.0)
**Safety**	9.0 (7.5–10.5)	81.8 (68.2–95.5)	9.5 (8.0–10.0)	86.4 (72.7–90.9)
**Pre-testing phase**	10.0 (8.0–11.0)	83.3 (66.7–91.7)	10.0 (9.0–11.0)	83.3 (75.0–91.7)
**Testing phase**	6.5 (5.0–7.5)	72.2 (55.6–83.3)	7.0 (6.0–8.5)	77.8 (66.7–94.4)
**Post-testing phase**	6.5 (5.5–8.0)	72.2(61.1–88.9)	6.5 (5.5–8.0)	72.2 (61.1–88.9)
EQA*	2 (0.0–3.0)	25.0 (0–37.5)	7.5 (6.0–8.0)	93.8 (75.0–100.0)
Overall score*	**40.5 (36.0–45.0)**	**64.1 (56.3–70.3)**	**49.0 (44.0–53.0)**	**76.6 (68.8–82.8)**

*Ranksum test p value (for differences between round-one and two) was < 0.05 for overall score, training and certification, and EQA. IQR: inter-quartile range; EQA: external quality assessment

The scores for EQA improved from a median score of 2 (out of 8) in the first-round assessment to a median score of 7.5 (out of 8) in the second-round assessment (Table 3). The median score for training and certification improved by 20% from a median score of 3 in round-one to a median score of 5 (out of a highest score of 10) in round-two. No improvement observed in certification of facilities as no nationally accredited certification programme exists for HIV rapid testing in South Africa. However, training coverage had increased which increased the score for this domain. As no certification programme exists, despite the improvement in training, the training and certification domain still had the lowest score (median percentage score of 50.0%) of the seven domains.

The median percentage scores for physical facility, safety, and testing improved by 10.0% (from 90.0% to 100.0%), 4.6% (from 81.8% to 86.4%), and 5.6% (from 72.2% to 77.8%), respectively, in round-two. The median percentage scores for pre-testing and post-testing stayed unchanged (at 83.3% for pre-testing and 72.2% for post-testing).

#### Distribution of implementation levels in second round assessment

**Fig 4:**
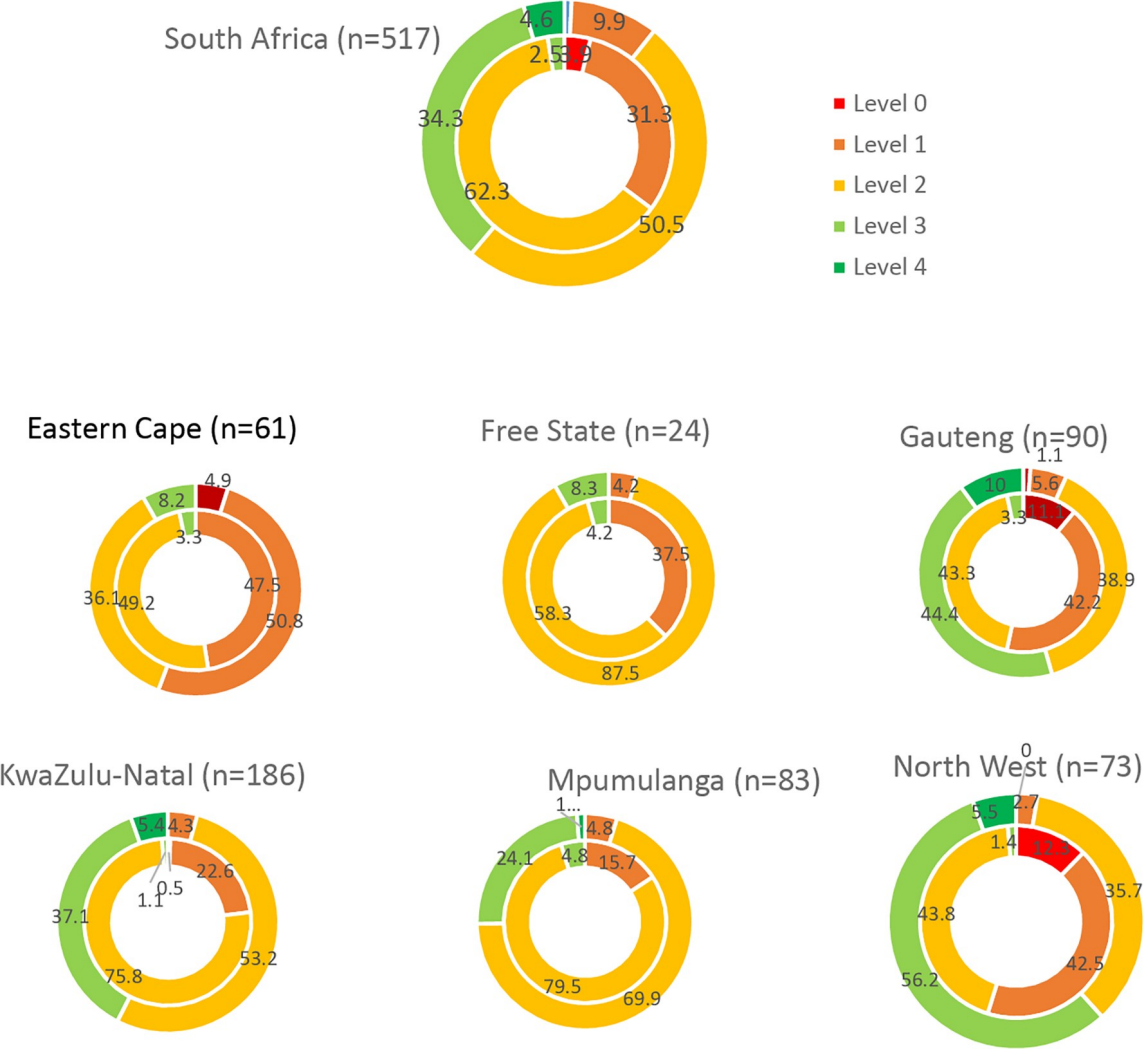
Distribution of implementation levels by assessment round (inner circle—Round 1; outer circle: Round 2) and province, SPI-RT assessment, 2015–2017, South Africa.

During round-two, 10.6% (55/517) of facilities were at level 1 or lower (with four facilities requiring improvement in all areas and immediate remediation); 50.5% (261/517) of facilities were partially ready for national certification (level 2); 34.3% (177/517) were close to national certification; and 4.6% (24/517) were eligible for national site certification (Fig 4 and S3 Fig).

The percentage of facilities at level 3 and above increased significantly from 2.5% (13/517) during round-one assessment to 38.9% (201/517) in round-two (p value from chi^2^ test = 0.001) (Fig 4). The largest improvements were seen in Gauteng and North West. In North West, 98.6% (72/73) of the facilities were at level 2 and below during round-one assessment, the number of facilities at level 2 and below substantially reduced to 38.4% (28/73) in round-two [61.6% (45/73) of facilities were at levels 3 and 4] (Fig 4).

#### Comparison of assessment result by province, assessment round and SPI-RT checklist domain

All eight provinces assessed showed improvement in training and certification and EQA domains. Two provinces (North West and Gauteng) improved their scores in all seven domains. In Eastern Cape, the scores for five of the seven domains (except EQA and training and certification) decreased (S6 Table).

#### Areas where facilities scored lowest and challenges documented

During the second assessment, the most common challenges documented were the lack of a national certification programme, unavailability of temperature control charts in the storage room for the test kits, infrastructural deficiencies (water supply out of order, no basin, no tap), infrequent IQC use [only 55.9% (289/517) of facilities used IQC routinely], use of previous testing algorithm, incorrect testing procedures, timer not available or inaccurately used, lack of knowledge on how to record invalid results, partial labelling of registers and lack of documentation (records) for competency assessment and corrective actions (Table 4).

**Table 4:** SPI-RT checklist question with the lowest score and challenges documented by domain, round-two conducted between 24 November 2016–29 September 17, SPI-RT assessment, South Africa (N = 517).

Domain	Questions with lowest scores	Percent of sites that scored 1 point	Most common challenges documented	Sites reporting challenges documented (n/N* (%)
Training	Have all testers been certified through a national certification programme?	0.6%	No national certification programme	245/245 (100.0)
	Are only certified testers allowed to perform HIV testing?	0.8%	No national certification programme	226/226 (100.0)
	Are all testers required to be re-certified periodically (e.g., every two years)?	0.9%	No national certification programme	226/226 (100.0)
	Are there records indicating all testers have demonstrated competency in HIV rapid testing prior to client testing?	25.0%	No competency assessment done	47/89 (52.8)
			No documentation of competency assessment	38/89 (42.7)
Physical facility	Are the test kits kept in a temperature-controlled environment based on the manufacturers’ instructions?	63.1%	No temperature control for test kits	46/84 (54.8)
	Is the testing area cleaned and organised for HIV rapid testing?	77.4%	Cluttered, dirty, untidy, unorganised	37/42 (88.1)
Safety	Are there clean water and soap available for hand-washing?	72.9%	Infrastructural deficiencies, water supply out of order, no basin, no tap water	69/76 (90.8)
			No hand soap	7/76 (9.2)
Pre-testing phase	Are test kits labelled with date received and initial?	41.8%	No date no initial	68/139 (48.9)
			Partial labelling (date only; labelled stock card only)	58/139 (41.7)
Testing phase	Are positive and negative quality control (QC) samples routinely used (e.g., daily or weekly) according to country guidelines?	55.9%	IQC not done according to the country guideline	38/114 (33.3)
			IQC done weekly only	41/114 (36.0)
			Not done consistently	20/114 (17.5)
	Are timers available and used routinely for HIV rapid testing?	65.2%	No timer	62/120 (51.7)
			Timer available but used inaccurately	38/120 (31.7)
			Timer available but not used routinely	14/120 (11.7)
			Timers have no batteries	4/120 (3.3)
	Are testing procedures adequately followed?	66.7%	Incorrect incubation time	32/112 (28.6)
			Incorrect drops	20/112 (17.9)
			Results read to early or to late	17/112 (15.2)
			Wrong testing procedures	18/112 (16.1)
Post-testing	Are invalid test results recorded in the register?	54.0%	Never had invalid result	7/21 (33.3)
			Invalid result not properly recorded	11/21 (52.4)
	Are invalid tests repeated and results properly recorded in the register or logbook?	47.8%	Invalid results repeated but not properly done and not recorded	44/84 (52.4)
			They are still following old algorithm	13/84(15.5)
	Are registers or logbooks properly labelled and archived when full?	48.3%	Full register not archived	22/83 (26.5)
			Register not labelled	25/83 (30.1)
EQA	Is an EQA/PT report received from National Reference Laboratory and reviewed by testers and/or the person in charge at the testing point?	59.4%	No records observed	11/56 (19.6)
			PT report not received	15/56 (26.8)
			Results not reviewed	19/56 (33.9)
	Does the testing point implement corrective action in case of unsatisfactory results?	60.7%	No corrective action despite low results	16/40 (40.0)
			No records of corrective action	7/40 (17.5)

* N: total number of sites reported /observed to experience at least one challenge for the domain assessed; n: sites that reported a specific challenge.

## Discussion

Monitoring of the RTCQI programme implementation in South Africa demonstrated improvement in the implementation of QA measures for HIV rapid testing. Among the 517 facilities assessed in both rounds, the percentage of facilities that were close to or eligible for national certification significantly increased from 2.5% during round-one assessment to 38.8% in round-two. Substantial improvement was achieved across provinces in the domains of EQA, and training and certification. The improvement in the EQA domain was mainly the result of the increase in participation in the nationally coordinated PT schemes (39.2% participated in PT in round-one assessment compared with 83.0% in the round-two assessment). However, result return rate could be improved further, as less than 90.0% of facilities returned result in all four PT cycles.

Even though the scores for training and certification domain improved (mainly as a result of progress in expanding training), the median percentage score for this domain remained low (at 50%) in round-two due to the lack of a national certification programme. There is no nationally agreed-upon set of criteria for competency and certification of staff in South Africa, which resulted in the lack of a national certification programme. A technical working group established in 2015 by NDoH made recommendations on criteria to be used for certifying testing providers [24]. By the end of 2018, these recommendations were yet to be implemented, thereby precluding high scores in that domain. Implementing a national certification programme requires adequate resources, infrastructure and personnel identified from independent organisations with demonstrated capacity to act as certifying bodies. The certification programme is useful as it reinforces the routine application of QA. It is also in line with current ISO 22870:2016 standards for point of care testing which emphasises three things to be intrinsic to the assessment for accreditation to ISO 22870: a) competence of testers, b) the assignment of competent QA manager(s) responsible for clinical governance and control of the QMS at site level, and c) the need for key control points such as audits, EQA and IQC.

The coverage of QA training was high: all selected facilities, representing 49.5% (2,077) of public facilities nationally, received QA training. However, the impact of the training on domains on which the training should have high impact (such as the pre-testing, testing, and post-testing domains) was moderate. The median percentage score for pre-testing (83.3%) and post-testing domains (72.2%) remained the same in both round-one and round-two assessments. The median score for testing domain increased by 5.6% (to 77.8%). The training followed a TOT approach. Representatives trained from each facility were to cascade training to all test providers within their designated facility. The extent to which trained facility staff cascaded the training to staff in their respective clinics is not known and this may have affected assessment two performance. The main gaps reported in the pre-test, test and post-test domains include infrequent use of IQC, incorrect testing procedures, inconsistent use of timer or unavailability of timer and incorrect recording. Consistent monitoring and follow-up of trained personnel through on-site supportive supervision is crucial, as sustaining the performance of trained test providers requires actions beyond just training. Underlying barriers at facility level (such as staff shortage and gaps in infrastructure) should be addressed by the relevant national and provincial government departments.

Substantial variation was observed across provinces in overall scores. North West, Eastern Cape and Gauteng provinces had the lowest scores in the round-one assessment; North West and Gauteng provinces significantly improved their scores in the round-two assessment. Eastern Cape showed no improvement in performance in round-two. Limpopo was already performing well in round-one. Lessons learned from the provinces that improved their performance in round-two and from those already performing well need to be shared with other provinces in order to strengthen QA programmes in all provinces. Differences in underlying health system factors (infrastructure, management and human resource) between provinces may contribute to the observed inter-provincial differences in performance.

To address underlying health system barriers, the South African government, as part of its commitment to quality health care, in a separate initiative is implementing the Ideal Clinic Realisation and Maintenance (ICRM) programme[25, 26]. The focus of the ICRM programme included infrastructure, adequate staff numbers, adequate medicines and supplies, administrative processes, and supply chain management. In addition, the ICRM would apply the necessary clinic policies and guidelines and work with partners and stakeholders to provide quality services. The ICRM manual includes participation in HIV PT and IQC use as part of its check list [27]. Additional health system strengthening at facility level include the clinic-laboratory interface (CLI) initiatives [28]. The ICRM is a comprehensive approach and has a potential to create an enabling environment for sustainable implementation of QA processes. However, coordination of the different initiatives (e.g. between RTCQI and ICRM on overlapping focus areas) is essential to achieve optimal results in improving quality at the facility level and to avoid duplication of effort.

Lack of documentation and routine use of IQC for corrective actions was documented as the main gap in the majority of facilities assessed. Despite the distribution of a large number (>10,000) of IQC panels, the number of facilities that routinely use IQC was low and IQC data were not collected and analysed centrally to monitor the routine use of IQC. In previous projects that NICD implemented, similar challenges were observed in IQC use. In 2013, after a four-year intensive implementation of training and monitoring of IQC use in Limpopo province, the coverage of IQC (i.e., facilities implementing IQC) was improved to 70%, but overall compliance to standards (among facilities using IQC) was below the acceptable range (less than 80.0%) [29]. Inappropriate storage of materials, lack of participation in training by staff performing testing, limited recording of data and data analysis were reported as key problems or barriers for improving routine use of IQC. It was noted that IQC programme implementation is highly staff and time intensive (i.e. monitoring IQC use requires conducting regular site visits and collecting and analysing IQC data) and requires the involvement of provincial staff to make sustainable progress in the implementation of the programme [29]. Other similar programme assessments done within the country also highlighted inconsistencies in IQC frequencies, stock outs, understaffing, and lack of QA officers as the main gaps in the QMS of the South African HIV rapid testing programme [12, 13, 17, 30, 31].

The following limitations of the study are acknowledged. RTCQI programme was implemented in only the 32 high-burden districts. Thus, results are not representative of all 52 districts in the country. However, because the facilities included from the 32 districts represent 90% HIV patient volume nationally, results can be generalised at the national level for high burden geographical areas. The study did not include low volume facilities. Low volume facilities may be at risk of poor performance due to relative under testing in these facilities. Limited information was collected on site-level health system characteristics which limited further multivariable analysis of facility level characteristics that may be associated with poor performance [32]. Facilities assessed in round two were not randomly selected. This could introduce bias in the findings as the sample used for round-two assessment may not represent facilities sampled in round-one. Some challenges were encountered in compiling the various data sources for these data analyses. Standard procedures for data management were not followed in electronically capturing/uploading the data. As a result, facility names and facility IDs, which were unique identifiers for this analysis, had a number of typing errors and missing values, which made the matching of data sources complicated. We were able to match 80–85% of the data using facility names or facility IDs, the remaining15–20% of the data were matched manually. This manual matching may have introduced some bias, which may result in underestimation or over estimation of performance. In future, we recommend the SPI-RT and PT data to be captured in a pre-programmed spreadsheet (Microsoft Excel or Access) and standardized facility names to be used for capturing this data.

## Conclusions

Substantial progress has been achieved over the last few years in progressively rolling out the RTCQI initiative which resulted in high (100%) coverage of QA training and increased participation in PT (>95%) in the 32 high-burden districts. The high levels of adherence to the testing algorithm in the PT programme was encouraging. Despite this, the improvement observed in the domains pre-testing, testing and post-testing phases (which are the domains that ultimately affect the accuracy of diagnosis) was modest.

Strengthening the documentation and use of IQC data for corrective actions and continuous supportive supervision and feedback mechanism will improve performance in all domains. Given that IQC is performed frequently (at least weekly in each testing point), a feasible and more sustainable method for collecting the IQC data could be explored. One such solution could be the electronic capturing of IQC data into the HIV testing module of the TIER.Net, electronic patient management system for South Africa. The same action would be beneficial for PT as the current system used for PT is highly manual. Technology transfers through collaboration to support sustainable country-owned EQA programmes may assist in improving both the PT and IQC programmes [33].

Focal persons trained from each facility should be accountable to oversee compliance to standards at facility level and should transfer their knowledge and skill to other test providers. The establishment of a national certification programme can be a very effective way to ensure reliability and accuracy of test results and encourage continuous quality improvement for both test providers at point of care and the testing facilities.

Given the current inadequate implementation of QA measures, the feasibility of re-testing clients at ART initiation points (using the same validated testing algorithm) could be explored as an additional QA measure to minimize misdiagnosis due to random or systematic errors.

## Supporting information

S1 File SPI-RT checklist version 2. (PDF)

S2 File SPI-RT checklist version 3. (PDF)

S3 File Proficiency testing form. (DOCX)

S1 Table Roles and responsibilities for stakeholders in the quality assurance programme in South Africa. (DOCX)

S2 Table HIV status of PT panels distributed in each PT cycle. (DOCX)

S3 Table List of approved HIV rapid diagnostic kits currently used in South Africa. (DOCX)

S4 Table Differences between SPI-RT checklist versions 2 and 3. (DOCX)

S5 Table Distribution of test kits used by sites for each PT cycle. (DOCX)

S6 Table Trends analysis of median scores by domain and province. (DOCX)

S1 Fig Implementation timeline of QA interventions and assessments in South Africa. (DOCX)

S2 Fig Proportion of facilities trained by year of training and province, South Africa. (DOCX)

S3 Fig Percentage of HIV testing facilities with increased performance levels in round-two, South Africa. (DOCX)
