# Supplementary material for: An overview of the quality assurance programme for HIV rapid testing in South Africa: Outcome of a 2-year phased implementation of quality assurance program
Source: PLoS One. 2019 Sep 26;14(9):e0221906. doi: 10.1371/journal.pone.0221906 (PMC6762059; doi:10.1371/journal.pone.0221906)
Supplement: S1 File — (PDF) [file pone.0221906.s001.pdf]

the Stepwise Process for Improving the Quality of HIV Rapid Testing (SPI-RT) Checklist

Version 2.0

SPI-RT Checklist

	SECTION QUESTIONS	YES	PARTIAL	NO	Comment
**1.0**	**PERSONNEL TRAINING AND CERTIFICATION**				
1.1.	Have the providers received a comprehensive training on HIV rapid test?				
1.2.	Are the providers trained on the use of standardized registers/logbooks?				
1.3.	Are the providers trained on proficiency testing (PT) process?				
1.4.	Are the providers trained on quality control (QC) process?				
1.5.	Are the providers trained on safety and waste management procedures?				
1.6.	Are there signed records of all procedures read and understood by HIV rapid testing personnel?				
1.7.	Is periodic (i.e. every two years) HIV rapid test refresher training offered for testing personnel?				
1.8.	Is there evidence that providers received adequate, specific training prior to patient testing to ensure competence?				
1.9.	If there is a national certification program, are providers certified?				
1.10.	Are only certified providers allowed to perform testing?				
1.11.	Are certified providers required re-certifications periodically (i.e. every two years)?				
**2.0**	**PHYSICAL FACILITY**				
2.1.	Is there a designated area for HIV testing?				
2.2.	Is the testing area clean and organized for HIV rapid testing?				

2.3.	Is sufficient lighting available in the designated testing area?				
2.4.	Is there continuous power supply available (If the kits are required to be stored in a refrigerator)?				
2.5.	Is there sufficient and secure storage space for test kits and other consumables?				
**3.0**	**SAFETY**				
3.1	Are there SOPs and/or job aides in place to ensure that personnel know to implement safety practices (apron, gloves, etc.)?				
3.2	Are there SOPs and/or job aides in place on how to dispose of infectious and non-infectious waste?				
3.3	Are there SOPs and/or job aides in place to manage spills of blood and other body fluids?				
3.4	Are there SOPs and/or job aides in place to address occupational exposure to potentially infectious body fluids through a needle stick injury, splash or other sharps injury?				
3.5	Are appropriate safety gears (i.e. gloves, lab coats or aprons) available for the providers?				
3.6	Are appropriate safety gears (i.e. gloves, lab coats or aprons) consistently used by the providers?				
3.7	Is there clean water and soap available for hand washing?				
3.8	Is there an appropriate disinfectant (i.e. bleach, alcohol, etc.) available?				
3.9	Are sharps, infectious and non-infectious waste handled properly?				
**4.0**	**PRE-TESTING PHASE**				

4.1	Are there national testing guidelines specific to the program available at the testing site?				
4.2	Is the national testing algorithm being used?				
4.3	Are there SOPs and/or job aides in place for each HIV rapid test used in the testing algorithm?				
4.4	Are only MOH approved HIV rapid kits available for use?				
4.5	Are test kits stored according to manufacturer recommendations?				
4.6	Is there a process in place for inventory management (receiving and monitoring supplies, handling expired kits, etc.)?				
4.7	Are reagents used within expiration date (First Expired, First Out principle)?				
4.8	Are test kits labeled with date received, date opened, and initials?				
4.9	Is there a process in place for alternative testing algorithm in case of expired or shortage of test kit(s)?				
4.10	Are job aides on specimen collection available and posted at the facility?				
4.11	Are there sufficient supplies available for specimen collection (i.e. lancets, gauze, alcohol swabs, plaster, etc.)?				
4.12	Are sharps (e.g., lancets and needles) disposed into appropriate containers after the finger prick procedure is performed?				
**5.0**	**TESTING PHASE**				
5.1	Are job aides on HIV testing procedures available and posted at the testing site?				
5.2	Are timers available and used routinely for HIV rapid testing?				
5.3	Are test devices properly labeled with client ID during testing?				

5.4	Are sample collection devices (capillary tube, loop, disposable pipettes etc.) used accurately?				
5.5	Are testing procedures adequately followed (during observation)?				
5.6	Are positive and negative quality control (QC) specimens routinely used (i.e. daily or weekly) according to country guidelines?				
5.7	Is QC results properly recorded?				
5.8	Are appropriate steps documented and taken when QC results are incorrect and/or invalid?				
5.9	Are QC records reviewed by a head of facility/testing site routinely?				
**6.0**	**POST-TESTING PHASE**				
6.1	Are test results properly recorded in register/logbook?				
6.2	Are test devices disposed of properly after testing?				
6.3	Is testing area properly cleaned and disinfected?				
6.4	Are waste containers emptied regularly?				
**7.0**	**DOCUMENTS AND RECORDS**				
7.1	Is there a national standardized HIV rapid testing register/logbook available and in use?				
7.2*	Are all the elements in the HIV rapid testing logbook/register captured correctly (i.e., kit names, lot numbers, expiration dates, client demographics, tester name, individual and final HIV results, etc.)?				
7.3	Are invalid test results recorded in the register/logbook, and then repeated?				

7.4*	Is the end of each page total summary for the register/logbooks complied accurately?				
7.5	Are all registers/logbooks and other documents kept in a secure location?				
7.6	Are registers/logbooks properly labeled and archived when full?				
7.7	Does the testing site ensure confidentiality of client information throughout all phases of the testing process?				
**8.0**	**EXTERNAL QUALITY ASSESSMENT (PT, RETESTING AND SITE SUPERVISION)**				
8.1	Is the testing site enrolled in an EQA/PT program?				
8.2	Do all providers at the testing site participate in the EQA/PT program?				
8.3	Does head of facility or testing review EQA/PT results before submission to NRL?				
8.4	Is EQA/PT feedback report received and reviewed?				
8.5	Does the site implement corrective action in case of unsatisfactory results?				
8.6*	Does the site collect samples for retesting (i.e. collection of DBS every 20th client)?				
8.7*	Are DBS samples collected properly (i.e., at least 3 complete circles)				
8.8*	Are DBS samples stored properly (i.e., away from sun light, separated by glassine paper, desiccant, etc.)				
8.9*	Are the IDs of DBS samples sent for retesting recorded?				
8.10*	Are the DBS results, upon receipt from NRL, recorded in the HIV register/logbook?				

8.11*	Does the site receive periodically supervisory team?				
8.12*	Is a direct observation of client testing performed during site supervision?				
8.13	Are providers retrained during site supervision?				
