# Supplementary material for: An overview of the quality assurance programme for HIV rapid testing in South Africa: Outcome of a 2-year phased implementation of quality assurance program
Source: PLoS One. 2019 Sep 26;14(9):e0221906. doi: 10.1371/journal.pone.0221906 (PMC6762059; doi:10.1371/journal.pone.0221906)
Supplement: S2 File — (PDF) [file pone.0221906.s002.pdf]

Stepwise Process for Improving the Quality of HIV Rapid Testing (SPI-RT) Checklist

SPI-RT Checklist

Version 3.0

10/16/2015

SPI-RT Checklist

PART A: CHARACTERISTICS OF THE FACILITY OR TESTING POINT AUDITED

Before completing the checklist, it is important to characterize the testing point to be audited. Please provide relevant information in the summary table below.

**Date of Audit** (dd/mm/yyyy)**:**	**Audit Round No:**
**Testing Facility Name:**	**Testing Facility ID** (if applicable)
**Testing Point Name:**	**Type of testing point** (Circle One)<br>**VCT/HTC** **PITC** **PMTCT** **TB clinic** **Laboratory**<br>**Treatment Center** **Other** (Specify)
**Location/Address:**	
**Level** (Circle One and specify name)<br>**Region/Province/Zone:**<br>**District:**<br>**Referral center:**<br>**Health center:**<br>**Dispensary:**<br>**Health Post:**<br>**Other** (Please specify to reflect country context):	**Affiliation** (Circle One)<br>**Government**<br>**Private**<br>**Faith-based Organization**<br>**Non-governmental organization**<br>**Other:**
**Number of Testers:**	**Average tested per month:**
**Name of the Auditor 1:**	**Name of the Auditor 2:**

PART B. SPI- RT Checklist

For each of the sections listed below, please check **Yes, Partial or No**, where applicable. Indicate "**Yes**" only when all elements are satisfactorily present. Provide comments for each "**Partial**" or "**No**" response. State N/A in the comments section if "not applicable" where appropriate (*).

SECTION		YES	Partial	NO	Comments	Score
**1.0**	**PERSONNEL TRAINING AND CERTIFICATION**					**10**
1.1	Have all testers received a comprehensive training on HIV rapid testing using the nationally approved curriculum?					
1.2	Are the testers trained on the use of standardized HIV testing registers/logbooks?					
1.3	Are the testers trained on external quality assessment (EQA) or proficiency testing (PT) process?					
1.4	Are the testers trained on quality control (QC) process?					
1.5	Are the testers trained on safety and waste management procedures and practices?					
1.6	Have all testers received a refresher training within the last two years?					
1.7	Are there records indicating all testers have demonstrated competency in HIV rapid testing prior to client testing?					
1.8	Have all testers been certified through a national certification program?					
1.9	Are only certified testers allowed to perform HIV testing?					
1.10	Are all testers required to be re-certified periodically (e.g., every two years)?					
**1.0**	**PERSONNEL TRAINING AND CERTIFICATION SCORE**					
**2.0**	**PHYSICAL FACILITY**					**5**
2.1	Is there a designated area for HIV testing?					

2.2	Is the testing area clean and organized for HIV rapid testing?					
2.3	Is sufficient lighting available in the designated testing area?					
2.4	Are the test kits kept in a temperature controlled environment based on the manufacturers' instructions?					
2.5	Is there sufficient and secure storage space for test kits and other consumables?					
**2.0 PHYSICAL FACILITY SCORE**						
**3.0 SAFETY**						**11**
3.1	Are there SOPs and/or job aides in place to implement safety practices?					
3.2	Are there SOPs and/or job aides in place on how to dispose of infectious and non-infectious waste?					
3.3	Are there SOPs and/or job aides in place to manage spills of blood and other body fluids?					
3.4	Are there SOPs and/or job aides in place to address accidental exposure to potentially infectious body fluids through a needle stick injury, splash or other sharps injury?					
3.5	Is personal protective equipment (PPE) always available to testers?					
3.6	Is PPE consistently used by all testers?					
3.7	Is PPE properly used by all testers through the testing process?					
3.8	Is there clean water and soap available for hand washing?					
3.9	Is there an appropriate disinfectant to clean the work area available?					
3.10	Are sharps, infectious, and non-infectious waste handled properly?					

3.11	Are infectious and non-infectious waste containers emptied regularly per the SOP and/or job aides?					
**3.0 SAFETY SCORE**						
**4.0 PRE-TESTING PHASE**						**12**
4.1	Are there national testing guidelines specific to the program (e.g. HTS, PMTCT, TB, etc.) available at the testing point?					
4.2	Is the national HIV testing algorithm being used?					
4.3	Is there a process in place for an alternative HIV testing algorithm in case of expired or shortage of test kit(s)?					
4.4	Are there SOPs and/or job aides in place for each HIV rapid test used in the testing algorithm?					
4.5	Are only nationally approved HIV rapid kits available for use currently?					
4.6	Are all the test kits currently in use within the expiration date?					
4.7	Are test kits labeled with date received and initials?					
4.8	Is there a process in place for stock management?					
4.9	Are job aides on client sample collection available and posted at the testing point?					
4.10	Are there sufficient supplies available for client sample collection?					
4.11	Are there national guidelines describing how client identification should be recorded in the HIV testing register?					
4.12	Are client identifiers recorded in the HIV testing register per national guidelines and on test devices?					
**4.0 PRE-TESTING PHASE SCORE**						
**5.0 TESTING PHASE**						9

5.1	Are SOPs and/or job aides on HIV testing procedures available and posted at the testing point?					
5.2	Are timers available and used routinely for HIV rapid testing?					
5.3	Are sample collection devices (e.g., capillary tube, loop, disposable pipettes, etc.) used accurately?					
5.4	Are testing procedures adequately followed?					
5.5	Are positive and negative quality control (QC) specimens routinely used (e.g., daily or weekly) according to country guidelines?					
5.6	Are QC results properly recorded?					
5.7	Are incorrect/invalid QC results properly recorded?					
5.8	Are appropriate steps taken and documented when QC results are incorrect and/or invalid?					
5.9	Are QC records reviewed by the person in charge routinely?					
**5.0 TESTING PHASE SCORE**						
**6.0**	**POST TESTING PHASE - DOCUMENTS AND RECORDS**					**9**
6.1	Is there a national standardized HIV rapid testing register/logbook available and in use?					
6.2	Does the HIV testing register/logbook include all of the key quality elements?					
6.3	Are all the elements in the register/ logbook recorded/captured correctly? (e.g., client demographics, kit names, lot numbers, expiration dates, tester name, individual and final HIV results, etc.)?					
6.4	Is the total summary at the end of each page of the register/logbooks complied accurately?					
6.5	Are invalid test results recorded in the register/logbook?					

6.6	Are invalid tests repeated and results properly recorded in the register/logbook?					
6.7	Are all client documents and records securely kept throughout all phases of the testing process?					
6.8	Are all registers/logbooks and other documents kept in a secure location when not in use?					
6.9	Are registers/logbooks properly labeled and archived when full?					
**6.0 POST TESTING PHASE - DOCUMENTS AND RECORDS SCORE**						
**7.0**	**EXTERNAL QUALITY AUDIT (PT, SUPERVISION AND RETESTING)**					**8/14**
7.1	Is the testing point enrolled in an EQA/PT program?					
7.2	Do all testers at the testing point test the EQA/PT samples?					
7.3	Does the person in charge at the testing point review the /PT results before submission to NRL or designee?					
7.4	Is an EQA/PT report received from NRL and reviewed by testers and/or the person in charge at the testing point?					
7.5	Does the testing point implement corrective action in case of unsatisfactory results?					
7.6	Does the testing point receive periodic supervisory visits?					
7.7	Is feedback provided during supervisory visit and documented?					
7.8	If testers need to be retrained, are they being retrained during the supervisory visit?					
**If the country external quality assessment program includes retesting of serum or DBS, proceed with questions 7.9 – 7.14. Otherwise, STOP here.**						
7.9*	Does the site collect samples for retesting according to country guidelines (e.g., collection of every 20th client serum or DBS sample)?					

7.10*	Are the serum or DBS samples collected for retesting properly documented?					
7.11*	Are serum or DBS samples collected properly (e.g., at least 3 complete circles or correct volume and correct tubes, etc.)?					
7.12*	Are serum or DBS samples stored properly (e.g., away from sunlight, separated by glassine paper, desiccant, or at 4oC or 20oC, etc.)?					
7.13*	Are the identifiers of serum or DBS samples sent for retesting properly recorded?					
7.14*	Are the serum or DBS results received from the referral lab properly documented and recorded in the HIV testing register/logbook?					
**7.0 EXTERNAL QUALITY AUDIT (PT, SUPERVISION AND RETESTING) SCORE**						

*Those marked with an asterisk are only applicable to sites where sample retesting is performed.

PART C: SCORING CRITERIA

Each element marked will be assigned a point value:

- Items marked “Yes” receive 1 point each.
- Items marked “Partial” receive 0.5 point each.
- Items marked “No” receive 0 point each.

Total points scored for each section should be tallied and recorded at the end of the section.

The overall total points obtained by each HIV testing point audited will be weighed to correspond to a specific performance level.

Levels	% Score	Description of results
Level 0	Less than 40%	Needs improvement in all areas and immediate remediation
Level 1	40% - 59%	Needs improvement in specific areas
Level 2	60%-79%	Partially eligible
Level 3	80%-89%	Close to national site certification
Level 4	90% or higher	Eligible to national site certification

Part D. Auditor's Summation Report for SPI-RT Audit

Facility Name:<br>Site Type:<br>Staff Audited Name:	No. of Tester(s):<br>Duration of Audit:	Total points scored (exclude N/A) = a<br>Total score expected = b<br>% Score = (a/b) x 100

Section No.	Deficiency/Issue observed	Correction Actions		Auditor's Comments	Recommendations	
		Immediate	Follow up		Actions	Timeline / Person responsible

Staff Audited Signature:<br><br>Person in Charge Name and Signature:	Auditor Name and Signature:<br><br>Date (dd/mm/yyyy):
