# Supplementary material for: An overview of the quality assurance programme for HIV rapid testing in South Africa: Outcome of a 2-year phased implementation of quality assurance program
Source: PLoS One. 2019 Sep 26;14(9):e0221906. doi: 10.1371/journal.pone.0221906 (PMC6762059; doi:10.1371/journal.pone.0221906)
Supplement: S3 File — (DOCX) [file pone.0221906.s003.docx]

## **S3 File:** Proficiency testing form


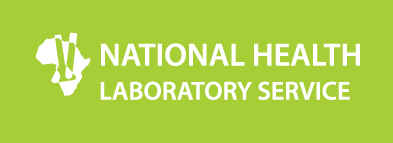
**NHLS Proficiency Testing Scheme - HIV Serology**

Practice Number: 5200296

**Participant Instructions**

**Your Identification Code is:**

1. **Introduction**

Thank you for participating in the HIV Serology Proficiency Testing Scheme (PTS). We hope that your participation will be beneficial to your facility, as well as enjoyable.

1. **Sample Details**

The package contains 6 samples (in Serum form) - **H01, H02, H03, H04, H05 and H06**

1. **Proficiency Test samples –Treatment and analysis**

**Samples should be treated in the same way as routine patient specimens.**

Samples should be stored between 2 – 8 ºC before testing, and must be tested using the routine rapid tests. Samples shall be tested using the national algorithm.

1. **Factors that could influence the testing i.e. Nature of the PT test samples, conditions of storage**

**Temperature:** Samples are shipped at Room Temperature. Please ensure that samples are stored at 2-8ºC upon receipt and at -20ºC for long term storage.

1. **Sample reception, Handling instructions and Safety requirements.**

Please follow universal safety precautions at all times.

Please open package and inspect for any breakages, possible deterioration during transportation or missing samples.

Leaking and broken samples should be disposed of immediately and repeat/replacement samples can be requested from NHLS within 5 working days.

1. **Recording and submission of PTS Results.**

Result forms must be completed in full and **Identification Code** must be **filled in and the form signed off by the Facility Manager.**

**The closing date for submission of results is 15 June 2017**

**NHLS PROFICIENCY TESTING SCHEME**

**HIV SEROLOGY**

**Please make sure all details are completed.**

| **Identification Code:** |  | **Survey number** | **0117** | **Signature (Facility Manager)** |
| --- | --- | --- | --- | --- |
| **Receipt Date of PT Samples:** |  | **Date of Testing PT samples:** |  |  |
| **PT Samples Tested by:** |  | **Facility Telephone No:** |  |  |

|  | **HIV TEST KIT USED** | **BATCH NUMBER** | **EXPIRY DATE** |
| --- | --- | --- | --- |
| **Test 1** |  |  |  |
| **Test 2** |  |  |  |

**PLEASE INDICATE WITH ☑ FOR FINAL INTERPRETED RESULTS.**

| **SAMPLE NUMBER** | **H01** | | **H02** | | **H03** | | **H04** | | **H05** | | **H06** | |
| --- | --- | --- | --- | --- | --- | --- | --- | --- | --- | --- | --- | --- |
| **KIT** | **Test 1** | **Test 2** | **Test 1** | **Test 2** | **Test 1** | **Test 2** | **Test 1** | **Test 2** | **Test 1** | **Test 2** | **Test 1** | **Test 2** |
| **INTERPRETATION** | □Non-Reactive □Reactive | □Non-Reactive □Reactive | □Non-Reactive □Reactive | □Non-Reactive □Reactive | □Non-Reactive □Reactive | □Non-Reactive □Reactive | □Non-Reactive  □Reactive | □Non-Reactive □Reactive | □Non-Reactive □Reactive | □Non-Reactive □Reactive | □Non-Reactive □Reactive | □Non-Reactive □Reactive |
| **FINAL RESULT** | □Positive □Negative | | □Positive □Negative | | □Positive □Negative | | □Positive □Negative | | □Positive □Negative | | □Positive □Negative | |

**N.B PLEASE GIVE US FEEDBACK BY FILLING IN SECTION BELOW!**

**Please answer the following questions by ticking the appropriate box and commenting in the space provided:**

| **QUESTIONS** | **YES** | **NO** |
| --- | --- | --- |
| 1. **Did you receive PTS samples in a good condition?** |  |  |
| 1. **Was the security seal still intact?** |  |  |
| 1. **Did you receive your previous PT report timeously?** |  |  |
| 1. **Was the report easy to interpret the previous report?** |  |  |
| 1. **Was your query/complaint handled in a satisfactorily manner?** |  |  |
| **Please comment in detail if you have ticked NO to any question between 1-5 or have a complaint/compliment/suggestion** | | |
