# Supplementary material for: An overview of the quality assurance programme for HIV rapid testing in South Africa: Outcome of a 2-year phased implementation of quality assurance program
Source: PLoS One. 2019 Sep 26;14(9):e0221906. doi: 10.1371/journal.pone.0221906 (PMC6762059; doi:10.1371/journal.pone.0221906)
Supplement: S1 Table — (DOCX) [file pone.0221906.s004.docx]

## **S1 Table:** Roles and responsibilities for stakeholders in the quality assurance programme in South Africa

| **Entity** | **Role and responsibility*** |
| --- | --- |
| National Department of Health | Endorsement of implementation plan |
|  | Overall oversight of programme |
|  | HCT Policy endorsement |
|  | Certification of trainers and trainees |
|  | Guideline dissemination |
| National Laboratory Technical Working Group | Policy development |
|  |  |
|  | Reviewing the key reference documentation for HIV testing services (HTS) |
|  | Quarterly review meetings |
|  | Policy and system development for Post Marketing Surveillance |
| Provincial Department of Health | Ownership of programme at provincial level |
|  | Ensure adequate budget for programme |
|  | Address/administer corrective actions that may be required including follow-up |
|  | Develop standard operating procedures (SOPs) and post marketing surveillance at the level of the depots to ensure correct receipt and storage of rapid diagnostic test (RDT) |
|  | Appoint quality assurance (QA) Officers to assist with implementation of QA Activities |
|  | Supervision of programme implementation at provincial level |
|  | Guideline dissemination |
| National Health Laboratory Services/National Institute of Communicable Diseases | Continued provision of technical assistance to the NDoH   - Policy and guideline development - Development and review of training materials |
|  | Operational oversight of programme |
|  | Development of an implementation strategy |
|  | Provision of training of trainers (TOT) and refresher QA courses |
|  | Preparation and distribution of internal quality control (IQC) samples and proficiency testing (PT) panels |
|  | Determine distribution schedule |
|  | Review of IQC log sheets and analysis of PT data |
|  | Review & sharing of data and findings with National technical working group (TWG) |
|  | Validation and verification of all HIV test kits |
|  | Post marketing surveillance of all approved HIV test kits |
|  | Monitoring and evaluation of the QA programme as well as provision of regular feedback to provincial and national department of health offices (DoHs) |
| Implementing Partners | Collaborate with the Provincial department of health (DoH) |
|  | Roll out and cascade of training to facility level |
|  | Provide technical support at facility level   - Development of site specific operational SOPs, Job Aids and Operational Workflows - Facility preparedness for participation in the QA programme - Implementation of corrective actions |
| Testing Sites | Identify point of contact (POC) for receipt of IQC samples and PT panels |
|  | Ensure all staff conducting HIV rapid testing are trained and certified |
| Centres for Disease Control and Prevention | Technical and financial support to programme implementation |

***** *Table taken from the national quality assurance–quality improvement plan for HIV rapid testing*
