# Supplementary material for: An overview of the quality assurance programme for HIV rapid testing in South Africa: Outcome of a 2-year phased implementation of quality assurance program
Source: PLoS One. 2019 Sep 26;14(9):e0221906. doi: 10.1371/journal.pone.0221906 (PMC6762059; doi:10.1371/journal.pone.0221906)
Supplement: S2 Table — (DOCX) [file pone.0221906.s005.docx]

## **S2 Table:** HIV status of PT panels distributed in each PT cycle

| PT cycle | PT panel 1 | PT panel 2 | PT panel 3 | PT panel 4 | PT panel 5 | PT panel 6 |
| --- | --- | --- | --- | --- | --- | --- |
| 116 (round 1) | Non-reactive | Reactive | Reactive | Reactive | Non-reactive | Non-reactive |
| 216 (round 2) | Reactive | Reactive | Reactive | Non-reactive | Non-reactive | Non-reactive |
| 117 (round 3) | Non-reactive | Reactive | Non-reactive | Non-reactive | Reactive | Non-reactive |
| 217 (round 4) | Reactive | Non-reactive | Non-reactive | Reactive | Reactive | Non-reactive |
