# Supplementary material for: An overview of the quality assurance programme for HIV rapid testing in South Africa: Outcome of a 2-year phased implementation of quality assurance program
Source: PLoS One. 2019 Sep 26;14(9):e0221906. doi: 10.1371/journal.pone.0221906 (PMC6762059; doi:10.1371/journal.pone.0221906)
Supplement: S3 Table — (DOCX) [file pone.0221906.s006.docx]

S3 Table: List of approved HIV rapid diagnostic kits currently used in South Africa

| Province | First-line assay | Second-line assay |
| --- | --- | --- |
| Eastern Cape | One Step Anti-HIV 1&2 | Bio Tracer HIV1/2 |
| Free State | One Step Anti-HIV 1&2 | Bio Tracer HIV1/2 |
| Gauteng | ABON HIV 1/2/0 | First Response HIV 1-2-0 |
| KwaZulu-Natal | One Step Anti-HIV 1&2 | Bio Tracer HIV1/2 |
| Limpopo | ABON HIV 1/2/0 | First Response HIV 1-2-0 |
| Mpumalanga | ABON HIV 1/2/0 | First Response HIV 1-2-0 |
| Northwest | Toyo Anti-HIV 1/2 | First Response HIV 1-2-0 |
| Northern Cape | Toyo Anti-HIV 1/2 | Bio Tracer HIV1/2 |
