# Supplementary material for: An overview of the quality assurance programme for HIV rapid testing in South Africa: Outcome of a 2-year phased implementation of quality assurance program
Source: PLoS One. 2019 Sep 26;14(9):e0221906. doi: 10.1371/journal.pone.0221906 (PMC6762059; doi:10.1371/journal.pone.0221906)
Supplement: S4 Table — (DOCX) [file pone.0221906.s007.docx]

S4 Table: Differences between SPI-RT checklist versions 2 and 3

| Domains | Total number of questions** | |
| --- | --- | --- |
|  | **Version 2** | **Version 3** |
| Personnel and certification | 11 | 10 |
| Physical facility | 5 | 5 |
| Safety | 9 | 11 |
| Pre-testing phase | 12 | 12 |
| Testing phase | 9 | 9 |
| Post-testing phase | 4 | 9 |
| Documents and records | 7 | Not applicable* |
| EQA | 8 | 8 |
| Overall | **65** | **64** |
| General information: Average monthly testing | Not included | Included |

** Version 2 had eight domains. In version 3, post-testing and documentation were combined into one domain, giving a total of seven domains in version 3.*

*** The maximum score that could be given for each question was “1”;thus the highest possible score that can be achieved per domain was equal to the total number of questions in each domain.*
