# Supplementary material for: An overview of the quality assurance programme for HIV rapid testing in South Africa: Outcome of a 2-year phased implementation of quality assurance program
Source: PLoS One. 2019 Sep 26;14(9):e0221906. doi: 10.1371/journal.pone.0221906 (PMC6762059; doi:10.1371/journal.pone.0221906)
Supplement: S5 Table — (DOCX) [file pone.0221906.s008.docx]

|  |  | 2016 | | 2017 | | |
| --- | --- | --- | --- | --- | --- | --- |
| Type of test | **Test brand name** | **Cycle 1*** | **Cycle 2*** | **Cycle 1*** | **Cycle 2**** |  |
| Screening test kit | Advanced Quality anti HIV 1&2 | 83.5% | 90.4% | 89.3% | 78.3% |  |
|  | Determine, Unigold | 0.3% | 0.1% |  |  |  |
|  | ABON HIV 1/2/O Tri-Line |  | 0.1% | 0.2% | 10% |  |
|  | KHB Rapid HIV 1/2 |  | 0.1% |  |  |  |
|  | First Response HIV 1-2-O |  |  | 0.1% | 0.1% |  |
|  | One Step Anti-HIV 1/2 Tri-Line |  |  |  | 3.3% |  |
|  | Not specified | 16.4% | 9.3% | 10.4% | 8.3% |  |
| Confirmatory test kit | ABON HIV 1/2/O Tri-Line | 83.3% | 89.8% | 89.6% | 76.2% |  |
|  | First Response HIV 1-2-O | 0.3% | 0.1% |  | 11.0% |  |
|  | Advanced Quality anti HIV 1&2 |  | 0.1% |  | 0.1% |  |
|  | One Step Anti-HIV 1/2 Tri-Line |  |  |  | 0.2% |  |
|  | Not specified | 16.4% | 10.0% | 10.4% | 12.6% |  |

S5 Table: Distribution of test kits used by sites for each PT cycle

* National algorithm combined Advanced Quality Rapid Anti-HIV (1&2) for screening and ABON HIV 1/2/O Tri-Line for confirmatory.

** The following new algorithm was introduced in 2017 2^nd^ cycle: ABON and First Response; or One Step Anti-HIV 1&2 & Bio Tracer HIV1/2; or Toyo Anti-HIV 1/2 & Bio Tracer HIV1/2; or Toyo Anti-HIV 1/2 & First Response
