# Supplementary material for: An overview of the quality assurance programme for HIV rapid testing in South Africa: Outcome of a 2-year phased implementation of quality assurance program
Source: PLoS One. 2019 Sep 26;14(9):e0221906. doi: 10.1371/journal.pone.0221906 (PMC6762059; doi:10.1371/journal.pone.0221906)
Supplement: S6 Table — (DOCX) [file pone.0221906.s009.docx]

S6 Table: Trends analysis of median scores by domain and province

| **Province** | **Phase** | **Training & Certification**  **(%) (IQR)** | **Physical facility (%) (IQR)** | **Safety**  **(%) (IQR)** | **Pre-testing phase**  **(%) (IQR)** | **Testing phase**  **(%) (IQR)** | **Post-testing phase**  **(%) (IQR)** | **EQA**  **(%) (IQR)** |
| --- | --- | --- | --- | --- | --- | --- | --- | --- |
| EC | 1^st^ round | 15.0  (10.0 – 40.0) | 100.0  (80.0 – 100.0) | 81.8  (63.6 – 90.9) | 70.8  (62.5 – 83.3) | 66.6  (55.6- 83.3) | 66.6  (50.0 –72.2) | 25.0  (0.0- –37.5) |
|  | 2^nd^ round | 40.0  (30.0- 45.0) | 80.0  (70.0 – 100.0) | 68.2  (50.0 – 81.8) | 58.3  (50.0- 75.0) | 61.1  (44.4 – 88.9) | 44.4  (33.3 – 66.7) | 75.0  (50.0 – 87.5) |
| FS | 1^st^ round | 25.0  (20.0 – 35.0) | 90.0  (80.0 –100.0) | 79.5  (63.6– 81.8) | 83.3  (77.1 – 87.5) | 72.2  (50.0 – 83.3) | 61.1  (55.6 – 75.0) | 28.1  (3.1 – 40.6) |
|  | 2^nd^ round | 40.0  (30.0- 47.5) | 85.0  (75.0 – 100.0) | 84.1  (72.7 – 88.6) | 83.3  (75.0 – 87.5) | 77.8  (55.6 – 86.1) | 55.6  (38.9 – 69.4) | 100.0  (93.8 – 100.0) |
| GP | 1^st^ round | 20.0  (0.0 – 25.0) | 100.0  (80.0- 100.0) | 77.3  (63.6 – 90.9) | 75.0  (66.7 – 83.3) | 75.0  (44.4- 88.9) | 66.7  (50.0 – 88.9) | 25.0  (12.5 – 37.5) |
|  | 2^nd^ round | 55.0  (45.0 – 70.0) | 100.0  (90.0 – 100.0) | 90.9  (81.8 – 100.0) | 91.7  (79.2 – 95.8) | 80.6  (61.1 – 94.4) | 77.8  (72.2 – 88.9) | 87.5  (68.8 – 100.0) |
| KZN | 1^st^ round | 30.0  (20.0 – 35.0) | 100  (80 – 100) | 95.5  (86.4 – 100.0) | 83.3  (79.2- 91.7) | 72.2  (50.0- 83.3) | 77.8  (66.7 – 88.9) | 12.5  (0.0 – 37.5) |
|  | 2^nd^ round | 50.0  (40.0 – 60.0) | 100  (80 – 100) | 90.9  (81.8 – 95.5) | 83.3  (75.0 – 91.7) | 83.3  (66.7 – 94.4) | 72.2  (61.1 – 83.3) | 100.0  (75.0 – 100.0) |
| MP | 1^st^ round | 30.0  (20.0 – 40.0) | 90  (80- 100) | 81.8  (72.7 – 90.9) | 87.5  (79.2 – 95.8) | 77.8  (66.7 – 88.9) | 88.9  (77.8 – 94.4) | 25.0  (12.5 –37.5) |
|  | 2^nd^ round | 50.0  (40.0 – 55.0) | 80.0  (70.0 – 90.0) | 72.7  (68.2 – 81.8) | 87.5  (79.2 – 91.7) | 77.8  (66.7 – 88.9) | 72.2  (66.7 – 83.3) | 100  (87.5 – 100.0) |
| NW | 1^st^ round | 55.0  (40.0 –65.0) | 90.0  (70.0 – 100.0) | 63.6  (50.0 – 72.7) | 58.3  (50.0 – 75.0) | 77.8  (44.4 – 88.9) | 72.2  (50.0 – 88.9) | 12.5  (0.0 – 28.6) |
|  | 2^nd^ round | 60.0  (45.0- 65.0) | 100.0  (90.0 – 100.0) | 90.9  (81.8 – 95.5) | 91.7  (83.3 – 95.8) | 83.3  (77.8 – 94.4) | 83.3  (77.8 – 94.4) | 87.5  (75.0 – 100.0) |
| All | 1^st^ round | 30.0  (20.0 – 40.0) | 90.0  (80.0 –100.0) | 81.8  (68.2 –95.5) | 83.3  (66.7 –91.7) | 72.2  (55.6 –83.3 | 72.2  (61.1 –88.9) | 25.0  (0.0 –37.5) |
|  | 2^nd^ round | 50.0  (40.0 – 60.0) | 100.0  (80.0 –100.0) | 86.4  (72.7 – 90.9) | 83.3  (75.0 –91.7) | 77.8  (66.7 –94.4) | 72.2  (61.1 –88.9) | 93.8  (75.0 –100.0) |
| p value ^*^ | | 0.0001 | 0.0001 | 0.0001 | 0.0001 | 0.0001 | 0.0001 | 0.0001 |

**Kruskall*–*Wallis test for differences in the distribution of scores by assessment round and provinces*
