# Supplementary material for: An overview of the quality assurance programme for HIV rapid testing in South Africa: Outcome of a 2-year phased implementation of quality assurance program
Source: PLoS One. 2019 Sep 26;14(9):e0221906. doi: 10.1371/journal.pone.0221906 (PMC6762059; doi:10.1371/journal.pone.0221906)
Supplement: S1 Fig — (DOCX) [file pone.0221906.s010.docx]

**S1 Fig:** Implementation timeline of QA interventions and assessments in South Africa

**Round-two assessment started on 24 Nov 2016**

**Assessment**

**Round-one assessment started**

**on 18 April 2015**

**IQC sample distribution started on 1 September 2014**

**QA training started on 3 April 2014**

**PT programme started on 18 May 2016**

**Intervention**
