# Supplementary material for: An overview of the quality assurance programme for HIV rapid testing in South Africa: Outcome of a 2-year phased implementation of quality assurance program
Source: PLoS One. 2019 Sep 26;14(9):e0221906. doi: 10.1371/journal.pone.0221906 (PMC6762059; doi:10.1371/journal.pone.0221906)
Supplement: S3 Fig — (DOCX) [file pone.0221906.s012.docx]

S3 Fig: Percentage of HIV testing facilities with increased performance levels in round-two, South Africa
